# Mass spectrometry analysis of tau and amyloid‐beta in iPSC‐derived models of Alzheimer’s disease and dementia

**DOI:** 10.1111/jnc.15315

**Published:** 2021-03-02

**Authors:** Charles Arber, Argyro Alatza, Claire A. Leckey, Ross W. Paterson, Henrik Zetterberg, Selina Wray

**Affiliations:** ^1^ Department of Neurodegenerative Disease UCL Queen Square Institute of Neurology University College London London UK; ^2^ Translational Mass Spectrometry Research Group UCL Great Ormond Street Institute of Child Health University College London London UK; ^3^ UK Dementia Research Institute at UCL London UK; ^4^ Department of Psychiatry and Neurochemistry Institute of Neuroscience and Physiology Sahlgrenska Academy at the University of Gothenburg Mölndal Sweden; ^5^ Clinical Neurochemistry Laboratory Sahlgrenska University Hospital Mölndal Sweden

**Keywords:** Alzheimer's disease, amyloid‐beta, induced pluripotent stem cells, mass spectrometry, tau

## Abstract

Induced pluripotent stem cell (iPSC) technology enables the generation of human neurons in vitro, which contain the precise genome of the cell donor, therefore permitting the generation of disease models from individuals with a disease‐associated genotype of interest. This approach has been extensively used to model inherited forms of Alzheimer's disease and frontotemporal dementia. The combination of iPSC‐derived neuronal models with targeted mass spectrometry analysis has provided unprecedented insights into the regulation of specific proteins in human neuronal physiology and pathology. For example enabling investigations into tau and APP/Aβ, specifically: protein isoform expression, relative levels of cleavage fragments, aggregated species and functionally critical post‐translational modifications. The use of mass spectrometry has enabled a determination of how closely iPSC‐derived models recapitulate disease profiles observed in the human brain. This review will highlight the progress to date in studies using iPSCs and mass spectrometry to model Alzheimer's disease and dementia. We go on to convey our optimism, as studies in the near future will make use of this precedent, together with novel techniques such as genome editing and stable isotope labelling, to provide real progress towards an in depth understanding of early neurodegenerative processes and development of novel therapeutic agents.

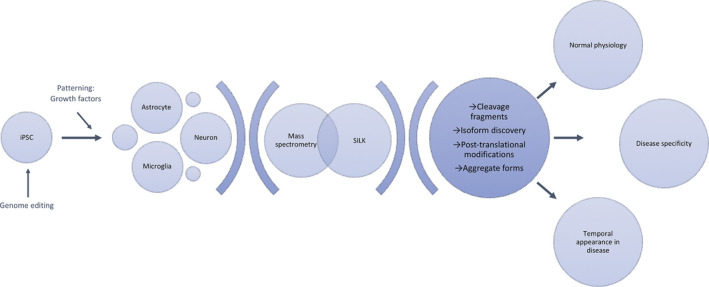

## Graphical abstract

This Review is part of the special issue ‘Mass Spectrometry in Alzheimer Disease’ and will highlight the progress to date in studies using induced pluripotent stem cells (iPSCs) and mass spectrometry to model Alzheimer's disease and dementia. iPSCs represent a limitless source of dementia‐associated cell types of interest. These cells contain the precise genome of the donor or can be manipulated with novel genome editing techniques, such as CRISPR/Cas9. iPSCs can then be differentiated into any somatic cell type using developmental principles and growth factor signalling that mimics developmental patterning cues. Untargeted and targeted proteomic analysis using mass spectrometry can provide unparalleled insight to the fragments, modifications, aggregations and isoforms observed for key peptides of interest. Additionally, SILK studies (stable isotope labelling kinetics), which measure the incorporation of heavy isotope labelled tracers in proteins over time and is dependent on mass spectrometry, allow for peptide turnover investigations. Together, these techniques and the understanding gained can help inform us on normal human brain physiology, disease‐specific signatures and a timeline of disease processes.

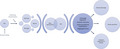

**Abbreviations**
AD: Alzheimer's disease
AGD: argyrophilic grain disease
APP: amyloid precursor protein
Aβ: amyloid β
CBD: corticobasal degeneration
CSF: cerebrospinal fluid
DS: down's syndrome
FTD: frontotemporal dementia
GGT: globular glial tauopathy
hESC: human embryonic stem cell
IP: immunoprecipitation
iPSC: induced pluripotent stem cell
LC‐MS/MS: liquid chromatography – tandem mass spectrometry
MALDI: TOF, matrix‐assisted laser desorption/ionization with time‐of‐flight
MAPT: microtubule‐associated protein tau gene
MS: mass spectrometry
PART: primary age‐related tauopathy
PiD: Pick's disease
PRM: parallel reaction monitoring
PSEN1: presenillin‐1
PSEN2: presenillin‐2
PSP: progressive supranuclear palsy
PTM: post‐translational modification
qPCR: quantitative polymerase chain reaction
RT‐PCR: reverse transcription polymerase chain reaction
SELDI‐TOF: surface‐enhanced laser desorption/ionization—time‐of‐flight
SILK: stable isotope labelling kinetics

## 1 ALZHEIMER'S DISEASE

Alzheimer's disease (AD) is characterized by two main pathologies: the extracellular deposition of Aβ peptides in amyloid plaques and the intracellular deposition of the tau protein in neurofibrillary tangles. Studies of autosomal‐dominant AD have placed amyloid upstream of tau, with causative mutations in the genes encoding the amyloid precursor protein (*APP*), presenilin‐1 (*PSEN1*) and presenilin‐2 (*PSEN2*) (Hardy, 2017). However, there is substantial evidence that tau plays a central role in AD pathogenesis. The extent of tau pathology correlates with symptom severity and the extent of neurodegeneration (Braak et al. 2003); tau knockout or reduction is protective against amyloid toxicity in pre‐clinical models (Gheyara et al. 2014; Ittner et al. 2010; Roberson et al. 2007; Vossel et al. 2010); and mutations in the tau gene, (*MAPT*) cause frontotemporal dementia (FTD) (Hutton et al. 1998; Poorkaj et al. 1998), confirming a causative link between tau dysfunction and neurodegeneration. Thus, understanding the two pathologies of AD and the molecular mechanisms linking them to neurodegeneration is an area of intense research. Pre‐clinical models recapitulating the main pathologies are key to this endeavour.

## 2 INDUCED PLURIPOTENT STEM CELL MODELS OF ALZHEIMER'S DISEASE

Although multiple in vitro and in vivo models of AD and FTD exist, it is only recently that induced pluripotent stem cell (iPSC) technology has enabled the generation of unlimited numbers of human neurons in the lab. Pluripotency can be induced in terminally differentiated cells such as fibroblasts by transduction with the four reprogramming factors cMYC, SOX2, OCT4 and KLF4. The resulting iPSC are indistinguishable from human embryonic stem cells (hESC) and can be differentiated into all three germ lineages (Takahashi et al. 2007).

Thus, by generating iPSC from a patient with a genotype and/or phenotype of interest and differentiating those cells into a disease‐relevant cell type, patient‐specific in vitro models containing the participant's precise genome can be used for pre‐clinical studies of disease mechanisms. This approach has revolutionized the in vitro modelling of neurodegenerative disease, by permitting a limitless in vitro supply of human neurons and glia with disease‐causing genes expressed at endogenous levels. The ability to generate distinct neuronal subtypes together with astrocytes and microglia from iPSC is particularly advantageous for diseases such as the tauopathies, where the pathophysiological characteristics are not confined to one specific cell type. In the tauopathies, both neurons and/or glial cells can display abnormal tau deposition, aggregates and /or hyperphosphorylated tau. For example primary age‐related tauopathy (PART) and ageing‐related tau astrogliopathy (ARTAG) are pure neuronal and astroglial tauopathies, respectively, whereas globular glial tauopathy (GGT), Pick's disease (PiD), progressive supranuclear palsy (PSP), corticobasal degeneration (CBD), argyrophilic grain disease (AGD) and Frontotemporal dementia with parkinsonism‐17 (FTDP‐17) present both neuronal and glial cell involvement (Crary et al. 2014; Kovacs et al. 2016; Ahmed et al. 2013; Buée & Delacourte, 1999; Sergeant et al. 2008; Sergeant et al. 1999; Yoshida, 2014; Togo et al. 2002; BRAAK & BRAAK, 1989; Yamada et al. 1992; Ferrer et al. 2019; Mackenzie & Neumann, 2016). Furthermore, the neuronal tau aggregates can be found in different brain regions depending on the disease: PSP and CBD display prominent tau pathology in the brain stem, for example while predominantly cortical tau pathology is present in AD. iPSC technology, therefore offers the opportunity for the specific cell type affected in disease to be generated in vitro in order to investigate the selective vulnerability of specific cell populations to pathology.

Although animal models have been widely used to gain mechanistic insights into AD, it has been particularly challenging to generate models that develop plaques, tangles and neurodegeneration within a single system, often requiring exogenous overexpression of multiple mutant genes (Oddo et al. 2003). This raises the possibility that human neurons are selectively vulnerable to AD‐associated pathologies. The use of rodent models has also been hampered by species‐specific features of the protein of interest, for example alternative splicing of the tau protein, which is complex, developmentally regulated and differs between rodents and humans (Goedert et al. 1989; Kosik et al. 1989; Takuma et al. 2003). Importantly, the lack of translation of Aβ and tau‐targeted therapeutics from pre‐clinical studies to clinical success supports the need for refined models. iPSC therefore provide an attractive approach to address this gap in our toolkit, acting as a human, physiologically relevant model to study the mechanism of a disease in the specific cell type which is selectively vulnerable in disease, and in the absence of exogenous gene expression.

Despite the advantages offered by iPSC, there are still some limitations associated with this model, particularly concerns around the maturity of differentiated cell types. iPSC are ‘rejuvenated’, that is the reprogramming process erases the cellular epigenetic signatures associated with donor age(Lee et al. 2020). Furthermore, multiple studies have shown that iPSC‐neurons closely resemble neurons, for example using transcriptomic comparisons (Patani et al. 2012). This must be taken into consideration when studying diseases such as AD where age is a major risk factor. This can now be overcome in part by using transdifferentiation to directly convert fibroblasts into neurons, bypassing an iPSC intermediate, which allows cells to retain epigenetic signatures of ageing (Mertens et al. 2015). Nonetheless, iPSC are the only means to obtain an unlimited supply of patient‐specific neurons and glia for the study of disease mechanisms, and permit the direct cellular consequences of disease‐associated mutations to be studied.

Progress made using iPSC technology to model AD and FTD linked to *MAPT* mutations is reviewed here (Arber et al. 2017; Lines et al. 2020). In this review, we focus specifically on insights gained from the use of mass spectrometry in conjunction with these models, and how this has enabled the precise measurement of peptide isoforms, cleavage fragments, multimeric species and post‐translational modifications.

## 3 MASS SPECTROMETRY IN ALZHEIMER'S DISEASE RESEARCH

Over the last few decades, mass spectrometry‐based analysis of Aβ has focused primarily on identification and quantification in CSF, plasma and brain tissue (Portelius, Bogdanovic, *et al*. 2010; Portelius *et al*. 2012; Wildburger *et al*. 2017; Nakamura *et al*. 2018). Mass spectrometry (MS) analytical strategies can vary considerably across studies, however, the most common approaches used for the analysis of Aβ so far have been matrix‐assisted laser desorption/ionization (MALDI) or surface enhancement laser desorption/ionization (SELDI) coupled to a time‐of‐flight (TOF) mass spectrometer as well as liquid chromatography‐tandem mass spectrometry (LC‐MS/MS). A mo

re detailed overview and technical comparison of these techniques for Aβ detection and quantification can be found here (Bros et al. 2015). MALDI‐TOF MS provides a sensitive, accurate and rapid method for the relative quantification of Aβ species. Targeted LC‐MS/MS approaches have commonly used electrospray ionization (ESI) in setups using high‐performance liquid chromatography (HPLC) or ultra performance liquid chromatography (UPLC) coupled to a triple quadrupole mass spectrometer, which offer high specificity, absolute quantification and multiplexing capabilities. A common feature between these approaches has been the need for enrichment of Aβ peptides during sample preparation, likely because of the low abundance of Aβ peptides in complex sample matrices. This has most commonly been achieved by immunoprecipitation (IP) using antibodies targeting the mid domain (4G8) and N‐terminus (6E10) of the Aβ peptide before MS analysis. Although IP‐MALDI‐TOF and SELDI‐TOF MS approaches offer relative quantification, they have played a pivotal role in identifying novel and truncated Aβ species, and with less system complexity and at lower costs than LC‐MS/MS (Lewczuk et al. 2004; Maddalena et al. 2004; Portelius et al. 2007).

Similar to Aβ, MS‐based approaches have played a critical role in the characterization of tau in AD research. In the human central nervous system there are six different tau isoforms that are produced and these are subject to extensive post‐translational modifications in both normal and disease conditions (Kametani et al. 2020; Morris et al. 2015). It is this vast heterogeneity of tau species that has posed a particular analytical challenge to mass spectrometrists. Furthermore, thorough characterization and quantification of tau proteoforms by MS has been further complicated by their low concentration in CSF. Therefore, profiling of tau species in CSF has previously required the use of well‐established tau antibodies to enrich for specific isoforms/phosphorylation sites by IP before subsequent MS‐based analysis (Portelius et al. 2008). In the last decade the introduction of quadrupole‐Orbitrap hybrid mass spectrometers has allowed for parallel reaction monitoring (PRM) strategies to be developed; utilizing the high resolution and high mass accuracy capabilities of these instruments these methods offer a targeted approach that has high selectivity, high sensitivity and multiplexing capabilities (Gallien et al. 2012; Peterson et al. 2012). It is these PRM‐based strategies in particular that have most recently advanced our understanding and interpretation of tau truncation and metabolism in AD both in vivo and in vitro (Barthélemy et al. 2016, 2019, 2020; Sato et al. 2018).

## 4 Aβ GENERATION FROM APP

**FIGURE 1:**
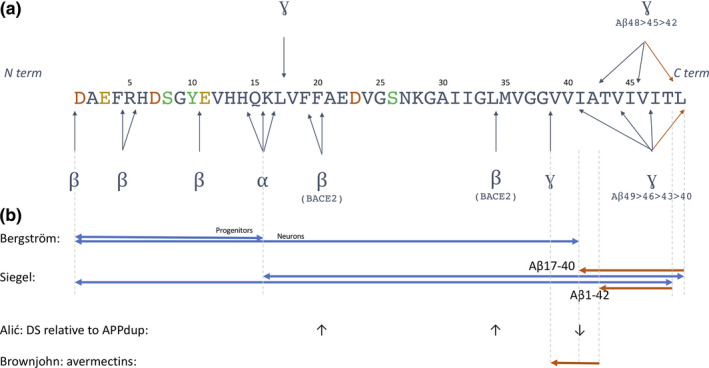
Amyloid‐beta sequence, cleavage sites and insights from mass spectrometry. (a) The amino acid sequence of Aβ, with residues labile to post‐translational modifications highlighted: Green—phosphorylation and dityrosine/nitrotyrosine/nitration of serine and tyrosine residues. Gold—pyroglutamate modification of glutamic acid residues. Red—racemization/isomerization of aspartic acid residues. Arrows show the sites of secretase cleavage of Aβ. (b) Information learned from iPSC models subject to mass spectrometry analysis. Predominant forms of Aβ in different stages of neuronal differentiation (Bergström et al. 2016). The finding that N terminal cleavage can direct C terminal endoproteolysis (Siegel et al. 2017). The anti‐amyloidogenic role of BACE2 in reducing relative amounts of amyloidogenic species in Down's syndrome neurons (Alić et al. 2020). The specificity of avermectins, increasing the processivity of γ‐secretase while leaving short species of Aβ unchanged (Brownjohn et al. 2017)

Canonical Aβ is produced when APP is sequentially cleaved by β‐secretase and γ‐secretase. However, there exists a spectrum of Aβ species that are produced when APP is cleaved by alternate enzymes, leading to truncations at both N and C termini (Figure 1). As such, Takami et al used a targeted LC‐MS/MS approach to show that γ‐secretase has C‐terminal truncating activity as the longest forms of Aβ (Aβ1‐49 and Aβ1‐48) are successively processed through parallel tripeptide cleavage pathways (Aβ1‐49 > 46>43 > 40 versus Aβ1‐48 > 45>42) to produce the most abundant fragments of Aβ (Takami et al. 2009).

In CSF, Aβ1‐40 is the most abundant species (Portelius et al. 2010). The relative levels of other C‐terminally truncated Aβ species such as Aβ1‐42 and Aβ1‐43 are prognostic for familial AD and diagnostic for amyloid plaque pathology in both familial and sporadic forms of the disease. These longer forms are a major constituent of pathological amyloid plaques, presumably because of increased hydrophobicity and enhanced aggregation. As a result the Aβ1‐42:Aβ1‐40 ratio in the CSF is used as a diagnostic test of AD (Janelidze et al. 2016).

The second most abundant form of Aβ is Aβ1‐17, which is also potentially β‐ and γ‐secretase‐dependent (Pérez‐Grijalba et al. 2014; Portelius et al. 2011). Shorter, non‐amyloidogenic isoforms ranging from Aβ1‐13 to Aβ1‐16 have been proposed to be α‐ and β‐secretase dependent (Portelius et al. 2011). Additionally, several Aβ species are produced by β‐secretase cleavage alone (BACE1 and BACE2 cleavage), producing species such as Aβ1‐19, Aβ1‐20 and Aβ1‐34 (Shi et al. 2003; Yan et al. 2001). Other enzymes (e.g. MMP2, MMP9 and Caspases) have been shown to be implicated in the generation of further Aβ species (e.g. Aβ1‐30) but are less well studied (Baranello et al. 2015).

N‐terminal truncation of Aβ includes cleavage at residues 2, 4, 5 and 11, each thought to be β‐secretase dependent (Vassar et al. 1999). Importantly, these N‐terminally truncated Aβ forms, such as Aβ3‐40/42 and Aβ11‐40/42, have been shown to be targets of post‐translational modifications such as pyroglutamate modification at glutamic acid 3 or 11; generating key amyloidogenic species (Mori et al. 1992; Saido et al. 1996). These modified Aβ forms are not secreted by neurons but appear to form in the tissue and may well be Aβ plaque‐specific.

Therefore, there is a huge diversity of Aβ species outside the well‐known Aβ1‐40 and Aβ1‐42. Mass spectrometry has been critical in detecting and distinguishing between various Aβ species in human brain tissue, CSF and plasma, and these approaches have recently been applied to iPSC models of AD.

## 5 Aβ DETECTION IN IPSC‐DERIVED NEURONS

To date, only a handful of studies, all using IP‐MALDI‐TOF MS, have investigated the entire spectrum of Aβ species produced in iPSC‐derived neurons (Bergström et al. 2016; Arber et al. 2020). Bergström and colleagues analysed the Aβ spectrum produced throughout iPSC differentiation; from the stem cell stage, through neural specification, to mature neuronal cultures (Bergström et al. 2016). APP expression and protein levels were found to be relatively consistent throughout differentiation, however, the processing of APP and the resultant Aβ species was tightly controlled in a cell stage‐specific manner. Short Aβ peptides, Aβ1‐15, Aβ1‐16 and Aβ1‐17, were primarily produced from days 10 to 25, at time points before the specification of neurons. This is consistent with predominant α‐secretase cleavage. From day 60 of differentiation onwards, after the generation of functional glutamatergic neurons (Kirwan et al. 2015; Shi et al. 2012), the majority of Aβ species produced were β‐ and γ‐secretase‐dependent, represented by Aβ1‐40, Aβ1‐42, Aβ1‐38 and Aβ1‐34. This study elegantly shows that Aβ is produced in a cell type and cell stage‐dependent manner, giving an insight into human developmental regulation of Aβ processing. β‐secretase expression (BACE1 and BACE2) and activity appear to be neuronally enriched, supporting the view that amyloidogenic Aβ is primarily generated within the neuronal population.

It has been shown that the Aβ spectrum produced from iPSC‐derived neurons is a representative model of the in vivo setting. Arber and colleagues showed that the most abundant species are Aβ1‐40, Aβ1‐17, Aβ1‐34, Aβ1‐38 and Aβ1‐19 (Arber et al. 2020), which is similar to the profiles in CSF. In addition to the expected C‐terminally processed Aβ species, representative N terminally truncated species were also detectable, including Aβ2‐x, Aβ4‐x, Aβ5‐x and Aβ11‐x (Arber et al. 2020).

iPSC‐derived neuronal models contributed to a detailed analysis of the N‐terminal truncation of Aβ and the downstream consequence on C‐terminal processing. Siegel and colleagues show that when Aβ is cleaved by α‐secretase at residue 17, γ‐secretase effectively processes the C‐terminus to Aβ40 (Aβ17‐40). However, when the substrate for γ‐secretase is β‐secretase dependent (Aβ1‐x or Aβ11‐x), proportionally longer Aβ is produced (i.e. Aβ1‐42 or Aβ11‐42) (Siegel et al. 2017). Enrichment for the APP intracellular domain (C terminal to Aβ) by IP followed by MALDI‐TOF MS analysis showed that this effect was in part driven by different endoproteolytic cleavage sites of γ‐secretase on APP. α‐secretase cleaved substrates predisposed Aβ17‐49 (and the Aβ49 > 46>43 > 40 processing pathway) whereas β‐secretase cleaved substrates predisposed Aβ1‐48 or Aβ11‐48 (and the Aβ48 > 45>42 processing pathway), thereby helping to explain the differences in relative Aβ1‐42 production.

In sum, these studies help to show the value of iPSC models in recapitulating the in vivo spectrum of Aβ generation (Figure 1). Additionally, mechanistic insights can be gained, such as the work by Siegel et al, explaining how the balance of Aβ1‐40 and Aβ1‐42 is achieved.

## 6 MODELLING FAMILIAL ALZHEIMER'S DISEASE

Stem cell models of Aβ generation have primarily focused on the ratio of Aβ1‐42:Aβ1‐40 (for review see Arber et al., 2017). It is widely accepted that mutations in *PSEN1* reduce the processivity of γ‐secretase (Chávez‐Gutiérrez et al. 2012; Szaruga et al. 2017), increasing the relative production of Aβ1‐42 compared to Aβ1‐40. Indeed, this has been corroborated using mutant *PSEN1* iPSC models (Yagi et al. 2011; Woodruff et al. 2013; Duan et al. 2014; Mahairaki et al. 2014; Sproul et al. 2014; Koch et al. 2012; Moore et al. 2015; Arber *et al*. 2020; Kwart et al. 2019; Raja et al. 2016; Liu et al. 2014; Arber, Villegas‐Llerena, et al., 2019). Additionally, mutations around the γ‐secretase cleavage site in *APP* have been shown to have a similar effect in the Aβ1‐42:Aβ1‐40 ratio, although this is achieved via favouring of the Aβ48 > 45>42 tripeptide cleavage pathway (Arber *et al*. 2020; Kondo et al. 2013; Kwart et al. 2019; Moore et al. 2015; Muratore et al. 2014). Mutations around the β‐secretase cleavage site or additional copies of *APP* because of local genomic duplications or Down's syndrome increase the total production of Aβ (Alić et al. 2020; Chang et al. 2015; Israel et al. 2012; Kondo et al. 2013; Kwart et al. 2019; Moore et al. 2015; Raja et al. 2016; Shi, Kirwan, Smith, MacLean, et al., 2012).

These studies help to reinforce mechanistic studies of γ‐secretase in a human model of familial AD expressing APP and secretase enzymes at physiological levels. However, studies using MALDI‐TOF MS have been able to advance these mechanistic insights. Mutations affecting the extracellular domain of PSEN1 (Y115H, Y115C and the splicing mutation int4del) display a relative increase in production of short species Aβ1‐14, Aβ1‐15 and Aβ1‐16 (Moore et al. 2015; Arber *et al*. 2020). This effect is not shared by *PSEN1* mutations in other domains of the protein and so may be a direct consequence of mutations to this substrate‐binding domain (Takagi‐Niidome et al. 2015). The relative increase in short Aβ species suggests an overall reduction in γ‐secretase activity and a concomitant increase in β‐ and/or α‐secretase activity.

In the last decade, iPSC technology has enabled the generation of organoids (organ‐like three‐dimensional tissues). Brain organoids provide an in vitro model that recapitulates the development of the human brain in which multilayers of several cell lineages self‐assemble and create an architecture of embryonic human brain‐like compartmentalization (e.g. forebrain, midbrain and hindbrain) with complex neural networks (Lancaster et al. 2013; Lancaster and Knoblich 2014; Lancaster et al. 2017; Qian et al. 2016, 2018; Jo et al. 2016). Therefore, the investigation of tissue development and disease state could be studied in a more ‘physiological’ multicellular model rather than into a single‐dimensional cellular level. Gonzalez and colleagues successfully generated cerebral organoids from people affected with fAD (PSEN1 mutation), Down syndrome and Creutzfeldt–Jakob disease. Over time, pathological features of AD were observed in the cerebral organoids, including Aβ and tau aggregates (Gonzalez et al. 2018). Furthermore, using a different organoid generation protocol, Raja and colleagues were able to reduce amyloid and tau pathology using β‐ and γ‐secretase inhibitors treatment (Raja et al. 2016). Recently, the validity of cerebral organoids as a model for investigating AD disease was further confirmed when AD patient‐derived organoids with either APOE ε3/ ε3 or APOE ε4/ε4 mutations recapitulated increased levels of Aβ and phosphorylated tau (Zhao et al. 2020).

Relative Aβ generation was further investigated in iPSC‐derived cerebral organoid models of Down's syndrome (DS) (Alić et al. 2020). The presence of three copies of *APP* because of duplication mutations predispose familial Alzheimer's disease with complete penetrance. *APP* is located on chromosome 21, and so individuals with DS also carry three copies of the *APP* gene. However, in contrast to *APP* duplication carriers, people with DS have only a 70% likelihood of developing Alzheimer's disease, suggesting the presence of protective genes on chromosome 21. Alić and colleagues presented data to support an anti‐amyloidogenic role for the β‐secretase gene *BACE2*, a gene also present in three copies in DS. When analysing Aβ isoforms generated by cerebral organoids, there was an increase in Aβ1‐19 and Aβ1‐34 relative to amyloidogenic species (Aβ1‐40 and Aβ1‐42) in Down's syndrome organoids when compared to isogenic diploid cells and *APP* duplication organoids. This increased BACE2‐associated β‐secretase activity, therefore, reduces the relative amount of amyloidogenic Aβ species. The authors witnessed a concomitant reduction in disease‐like signatures in DS organoids compared to *APP* duplication lines, such as amyloid‐like immunocytochemical staining. Genetic reduction of *BACE2* to two copies in trisomic cells exacerbated these disease‐like phenotypes, supporting a protective role for BACE2. Together these data demonstrate the delicate balance of cleavage activity and the importance of investigating the entire Aβ spectrum in a physiological model.

Finally, mass spectrometry analyses of iPSC‐derived Aβ species can have a central role in drug discovery. Brownjohn and colleagues screened compounds capable of increasing the processivity of γ‐secretase in Down's syndrome and familial AD iPSCs, i.e. molecules capable of increasing the Aβ1‐38:Aβ1‐42 ratio (Brownjohn et al. 2017). Importantly, mass spectrometry was used to support the finding that the lead compounds, avermectins, increased the generation of Aβ1‐37 and Aβ1‐38 at the expense of Aβ1‐40 and Aβ1‐42 without affecting smaller, γ‐secretase‐independent Aβ species. This selectivity of lead compounds is highly desirable in putative therapeutic agents.

Together, these studies demonstrate the importance of a representative appreciation for the entire Aβ spectrum (Figure 1). Mass spectrometry analysis, together with iPSC technology, highlight the interdependence and the delicate balance of different enzyme cleavages of Aβ. An understanding of the Aβ spectrum and this enzymatic balance is crucial when analysing the effect of therapeutic drug candidates or inherited Alzheimer's disease‐linked mutations.

## 7 FUTURE DIRECTIONS

Future work to analyse Aβ species without immunoprecipitation will be informative, as new truncated species, unknown post‐translational modifications and multimeric/aggregated species may be defined in this way. The use of immunoprecipitation can lead to bias in the species analysed, for example certain post‐translational modifications can alter the binding of antibodies used in immunoprecipitation. Of particular interest, examples of modified Aβ include pyroglutamate‐modified species at glutamic acid residues 3 and 11 (Perez‐Garmendia & Gevorkian, 2013), phosphorylation at serine 8 and serine 26 (Kumar et al. 2011, 2013), nitration at tyrosine 10 (Kummer et al. 2011), racemization/isomerization at aspartic acids 1, 7, 23 and 26 (Warmack et al. 2019) and dityrosine/nitrotyrosine modifications also at tyrosine 10 (Al‐Hilaly et al. 2014) (Figure 1). To date, little information exists about the post‐translational modification of Aβ species derived from iPSC‐neurons and these investigations will be highly informative. For example pyroglutamate modifications might be co‐culture or cell state dependent, as transglutaminase activity may be glial cell derived and dependent on inflammatory conditions (Wilhelmus et al. 2016). Furthermore, Aβ modifications may emerge in a temporal manner that could be linked to disease severity, as recently shown for tau (Dujardin et al. 2020; Wesseling et al. 2020).

## 8 TAU

Hyperphosphorylated aggregates of the microtubule‐associated protein tau are a pathological hallmark of a diverse group of neurodegenerative diseases collectively termed as tauopathies. Alzheimer's disease (AD) is the most common tauopathy, although it is in fact a secondary tauopathy; tau is downstream of altered Aβ production as evidence by the genetics of fAD as described above. The most common primary tauopathies (where tau is the defining pathological feature) include progressive supranuclear palsy (PSP), corticobasal degeneration (CBD) and frontotemporal dementia linked to mutations in the *MAPT* gene that encodes tau (Guo et al. 2017). It was the discovery of *MAPT* mutations that confirmed a causative link between tau dysfunction and neurodegenerative disease, and highlighted the importance of understanding the mechanisms linking tau to neuronal demise (Hutton et al. 1998; Poorkaj et al. 1998).

Alternative splicing of *MAPT* results in six protein isoforms of tau in the human central nervous system, differing by the presence of 0, 1 or 2 repeats at the N‐terminus of the protein (0N, 1N or 2N), and 3 or 4 microtubule‐binding repeats at the C‐terminus (3R or 4R). The expression of precise ratios of tau isoforms is regulated developmentally and dysregulated in disease, which will be discussed further below. Tau is further complicated by its extensive post‐translational modification. The most heavily studied of these is phosphorylation (Hanger et al. 2009). The phosphorylation of tau plays an important role in regulating its function, with phosphorylation at specific residues favouring the detachment of tau from the microtubules. In disease, tau is hyperphosphorylated, and the aberrant phosphorylation of tau may drive its aggregation.

There are 84 serine, threonine and tyrosine residues in tau, and mass spectrometry has enabled the direct identification of at least 51 specific phosphorylation sites in both in vitro and in vivo pre‐clinical models, as well as directly in control and disease post‐mortem brain tissue (Hanger et al. 2007; Wray et al. 2008). Additionally, in vitro studies have enabled candidate kinases for many phosphorylation sites to be identified, including GSK3β, CDK5 and CK1 (Hanger et al. 2009). A comprehensive list of tau phosphorylation sites identified in different diseases together with the kinases able to mediate phosphorylation at specific sites can be found here: https://docs.google.com/spreadsheets/d/1hGYs1ZcupmTnbB7n6qs1r_WVTXHt1O7NBLyKBN7EOUQ/edit#gid=0

Beyond phosphorylation, tau is also the target of numerous other post‐translational modifications (PTMs) including ubiquitination, acetylation, nitration, glycosylation and cleavage (Morris et al. 2015). A thorough catalogue of 170 distinct tau PTMs has recently been described in control and disease tissue (Kametani et al. 2020), and tau PTM state can correlate with clinical severity and heterogeneity (Barthélemy et al. 2020; Dujardin et al. 2020; Wesseling et al. 2020). Thus, there is a diverse range of tau species existing within a neuron at any one time, and it is important to understand the range of tau species, their disease specificity and the temporal manner in which they appear in disease.

There are a limited number of studies that have used mass spectrometry to analyse tau in iPSC‐neurons, these are discussed below.

## 9 MASS SPECTROMETRY ANALYSIS OF TAU IN IPSC

Tau splicing is developmentally regulated, with only the smallest tau isoform (0N3R) expressed at foetal stages (Goedert et al. 1989; Hefti et al. 2018). Additionally, splicing is dysregulated in disease, particularly in a subgroup of tauopathies where excess 4R is observed, including PSP and CBD. Intronic and splice‐site mutations in and around exon 10 of *MAPT* cause an increase in 4R tau and are causative of FTD, confirming a causal link between disrupted tau splicing and neurodegeneration (Hutton et al. 1998). Tau splicing is also species‐specific, and rodent models do not recapitulate the human pattern of tau isoforms (Yu et al. 2009). There has therefore been a great deal of interest in studying tau splicing in human neurons generated from iPSC. A number of studies have now shown that 0N3R is the predominant isoform expressed by iPSC neurons. Variable levels of 4R tau isoforms appear at varying culture times, and dependent on differentiation protocol, as detected by techniques including RT‐PCR, qPCR and western blot (Beevers et al. 2017; Iovino et al. ,^2010^, ^2015^; Sposito et al. 2015). Mass spectrometry provides the opportunity for the unambiguous detection of tau isoforms by confirming the presence of isoform‐specific peptides at exon‐exon junctions.

Disruption of the nuclear membrane and nucleocytoplasmic transport was observed in iPSC‐neurons with the *MAPT* 10 + 16 and P301L mutations (Paonessa et al. 2019). As P301L resides within exon 10, which is only expressed in 4R isoforms, there is a requirement for 4R expression in order to have mutant protein present in the model. This was confirmed by MALDI‐TOF/TOF mass spectrometry, which identified 4R‐specific peptides from both the wild‐type (HV **P** GGGSVQIVYKPVDLSK) and mutant (HV **L** GGGSVQIVYKPVDLSK) alleles (Paonessa et al. 2019). This confirms the presence of 4R tau, but does not provide quantitative information on the stoichiometry of the different tau isoforms. Sato et al used quantitative proteomics to assess the levels of 4R tau in iPSC‐neurons and post‐mortem brain tissue via the detection of two 4R‐specific peptides, LDLSNVQSK (amino acids 282‐290) and HVPGGSVQIVYK (amino acids 299‐311) (Sato et al. 2018). As expected, the 4R tau signal in the brain was around 50% of the total tau signal, corresponding to equimolar amounts of 3R:4R. In contrast, very low levels of 4R peptide could be detected in iPSC‐neurons, suggesting that at 6 weeks of culture, levels of total tau are around 100‐fold less than in the brain. A similar approach was unable to identify peptides corresponding to 1N or 2N tau isoforms in iPSC‐neurons at 5 weeks of differentiation. Low levels of the 4R‐specific peptide were detected in comparison to tau peptides ubiquitously present in all isoforms, further confirming the predominance of 0N3R in iPSC‐neurons (Silva et al. 2016).

Further studies of the detailed quantification of altered splicing in neurons with FTD‐linked splicing mutations will be of great interest, to help decipher developmental regulation and disease‐associated dysfunction in naïve human neurons.

## 10 TAU POST‐TRANSLATIONAL MODIFICATION IN IPSC‐NEURONS

Tau phosphorylation is also developmentally regulated, with high phosphorylation in early development thought to be related to the requirement for dynamic remodelling of the microtubule network (Brion et al. 1993). Hyperphosphorylation of tau is observed across the tauopathies, and accurate determination of the sites of phosphorylation and the relative stoichiometry in control versus disease is important in inferring the presence of “pathological” tau in pre‐clinical models. The majority of studies using iPSCs to model tauopathy have examined phosphorylation at specific sites using phospho‐specific antibodies, although a few studies have directly identified sites of phosphorylation using mass spectrometry.

Although the direct identification of phosphorylation sites on tau by mass spectrometry is informative, it is important to note that many of the same phosphorylation sites occur in development, control adult brain tissue and disease states (Kametani et al. 2020). Multiple quantitative approaches to allow the stoichiometry of phosphorylation at particular sites have been developed and will enable future work to identify disease‐associated changes. FLEXI‐tau is a mass spectrometry‐based assay that quantifies the ratio of modified/unmodified peptides in a culture system, with a decrease in the levels of unmodified peptides indicative of PTMs within the peptide (Mair et al. 2016). Caterina‐Silva *et al*. used the FLEXI‐tau assay to investigate post‐translational‐modifications of tau in iPSC‐neurons with the A152T variant compared with controls after 5 weeks in culture (Mair et al. 2016; Silva et al. 2016). There is an inherent assumption in many studies that equal expression from both the mutant and the unaffected allele is occurring within cells. A152T neurons showed increased levels of total tau overall, as well as increased levels of mutant tau, with A152T tau accounting for ~56.6% of total tau. Modification at several known phosphorylation epitopes was increased in A152T neurons (S202/205, T231/S235 and S396/S404), suggesting an early increase in phosphorylation at disease‐associated epitopes in tau mutation neurons. The A152T variant creates a potential novel phosphorylation site in tau through the addition of an extra threonine residue, however, no modifications were observed on the peptide covering this region suggesting it is unlikely to be phosphorylated in iPSC‐neurons (Silva et al. 2016). Using an immunoprecipitation‐mass spectrometry (IP‐MS) approach, an independent study identified multiple phosphorylated tau peptides in control neurons, including pT212, pS214 and pT217, pS262 and pS356 (Sato et al. 2018).

## 11 TAU TURNOVER IN IPSC‐NEURONS

Stable isotope labelling kinetics (SILK) allows for the measurement of protein production and turnover rates using labelling with heavy essential amino acids (typically ^13^C_6_‐leucine), followed by mass spectrometry to distinguish labelled from unlabelled peptides. Heavy leucine will be incorporated into newly synthesized proteins during the labelling phase, and the relative amount of labelled protein will reduce over time because of degradation. A thorough review of SILK in neurodegeneration is provided here (Paterson et al. 2019). Sato and colleagues used this approach to understand tau turnover in iPSC‐neurons (Sato et al. 2018).

Interestingly, disease‐associated tau species appear to have an increased turnover. For example 4R tau had a shorter half‐life than 3R tau, and several tau peptides containing phosphorylation residues (T111/S113/T123, T212/214, T217, S262/T263 and S356/T361) were shown to have a faster turnover than their non‐phosphorylated counterparts (Sato et al. 2018), suggesting differential proteostasis of specific tau species. It is also intriguing to note that the half‐life of tau in iPSC‐neurons is much shorter than in human participants (6.74 ± 0.45 days vs. 23 ± 6.4 days respectively) (Sato et al. 2018). This indicates developmental changes in tau proteostasis and is concordant with the idea that protective mechanisms against protein aggregation may become less efficient during ageing.

Further insights into the regulation of tau have been obtained by the identification of secreted tau fragments in the conditioned media from iPSC‐neurons previously labelled with ^13^C_6_‐leucine. Specifically, N‐terminal fragments of tau were observed in cell culture media, but no peptides covering the C‐terminal region (incorporating the microtube‐binding repeats) were observed (Sato et al. 2018). Together with antibody epitope mapping, the cleavage site was suggested to be between residues 210–230 of tau. The levels of labelled fragments increased in the media 3 days after labelling stopped, suggesting the cleavage and secretion of this fragment is a regulated, physiological process (Sato et al. 2018). The work by Sato *et al*. helped to change our understanding of tau in AD; the increase in total‐tau and phosphorylated‐tau seen extracellularly in AD represents an active secretion of phosphorylated and non‐phosphorylated N‐terminal tau fragments from live neurons exposed to Aβ, not passive release from dying neurons (Zetterberg, 2018).

## 12 PROTEOMICS TO UNCOVER MECHANISM OF TAU‐LINKED NEURONAL DYSFUNCTION

In addition to targeted proteomics, untargeted proteomics can provide a global and unbiased insight into dysregulated cellular pathways, an approach commonly utilized for biomarker discovery and one that has successfully identified early metabolic changes in post‐mortem AD brain tissue (Johnson et al. 2020). Tau pathology progresses throughout the brain in a predictable and defined manner, and recent research has focussed on mechanisms by which tau may be transferred from cell to cell (Braak et al. 2003). The propagation of tau via exosomes has been implicated in this process. Podvin and colleagues used iPSC‐neurons overexpressing the repeat region of tau with the FTD‐linked *MAPT* mutations P301L and V337 M to determine whether the presence of mutant tau would affect the composition of exosomes (Podvin et al. 2020). Widespread alterations in the proteome of exosomes from neurons expressing mutant tau were observed, including almost 245 proteins in control exosomes that were absent in those isolated from tau mutant overexpressing neurons. Several proteins uniquely present in tau exosomes have previously been linked to AD, including ANP32A, a potential modulator of tau phosphorylation, and PEN2, which is a subunit of γ‐secretase. It would be interesting to further extend this work to iPSC‐neurons with tau mutations; examining the impact of full length, mutant tau at the endogenous level.

## 13 FUTURE DIRECTIONS

Although the number of studies using mass spectrometry to analyse tau in iPSC‐neurons is relatively small, they have revealed the potential to gain insight into the physiological regulation of tau in healthy neurons and disease models. Future studies will likely enable a comprehensive characterization of the full range of tau PTMs, as previously described for mouse and human tau (Kametani et al. 2020; Morris et al. 2015). The importance of mapping tau PTMs has been highlighted by recent studies: cryoEM has enabled the detailed visualization of disease‐specific tau structures, which may be influenced by PTM profile (Arakhamia et al. 2020). Furthermore, specific signatures of tau PTMs identified by mass spectrometry in brain tissue and CSF have recently been shown to correlate with clinical heterogeneity and disease severity in AD (Barthélemy et al. 2020; Dujardin et al. 2020; Wesseling et al. 2020). Thus, tau PTMs are directly related to clinical outcomes, and detailed profiling in iPSC‐neurons will be essential to determine the extent to which they recapitulate disease pathologies.

## 14 CONCLUSIONS

Induced pluripotent stem cell technology is now over a decade old. This technology has enabled ‘disease in a dish’ studies that provide a human model of the cell type affected by disease, expressing mutant genes at physiological levels. Indeed, the number of iPSC‐related studies into Alzheimer's disease and dementia has steadily risen over the last decade, highlighting the relevance of this tool.

Mass spectrometry used in concert with iPSC technology has been instrumental in showing that these pre‐clinical models do indeed effectively model the in vivo setting. For example Aβ and tau are processed in a developmentally regulated manner and mature neuronal cultures are able to generate appropriate species of tau and Aβ spectra. The stage is now set to further develop these models to gain a deeper understanding of molecular mechanisms of neurodegeneration and screen for novel therapeutic agents.

iPSC‐derived cultures are reductionist, providing simplified models of the brain. This enables a cell‐type‐specific analysis into the consequences of gene mutations. Additionally, these models are foetal, because of cellular reprogramming and epigenetic erasure. This allows underlying, constitutive effects of a mutation to be distinguished from late stage, complex neurodegenerative processes. What is more, models are continually evolving and the advent of 3D cerebral organoid techniques (Lancaster et al. 2013) allow cell behaviours to be investigated in a model more akin to the developing brain. Additionally, CRISPR/Cas9 genome editing enables complex genetic manipulations to be performed, such as combinations that are not typically present in nature (Cong et al. 2013; Mali et al. 2013).

Going forward, mass spectrometry will become instrumental in these studies (Figure 1). As well as employing global proteomic approaches that can effectively describe the consequences of inherited forms of dementia, detailed analysis of post‐translational modifications will further our understanding of the earliest events of neurodegeneration. Adaptations of these techniques, such as SILK provide further crucial information, for example into the kinetics of protein turnover.

In sum, the combination of iPSC and mass spectrometry techniques have already proven the value of modelling Alzheimer's disease in vitro. The time is now for detailed mechanistic insights into early neurodegenerative processes and real progress to be made with drug discovery platforms towards novel therapeutic agents.

## CONFLICTS OF INTEREST

HZ has served at scientific advisory boards for Denali, Roche Diagnostics, Wave, Samumed, Siemens Healthineers, Pinteon Therapeutics and CogRx, has given lectures in symposia sponsored by Fujirebio, Alzecure and Biogen, and is a co‐founder of Brain Biomarker Solutions in Gothenburg AB (BBS), which is a part of the GU Ventures Incubator Program. The other authors have no conflicts of interest to declare.
